# Identification of Eight Small Nucleolar RNAs as Survival Biomarkers and Their Clinical Significance in Gastric Cancer

**DOI:** 10.3389/fonc.2019.00788

**Published:** 2019-09-06

**Authors:** Xuning Wang, Maolin Xu, Yongfeng Yan, Yanshen Kuang, Peng Li, Wei Zheng, Hongyi Liu, Baoqing Jia

**Affiliations:** Department of General Surgery, Chinese PLA General Hospital, Beijing, China

**Keywords:** small nucleolar RNA, biomarker, gastric cancer, survival, risk signature

## Abstract

Gastric cancer is one of most common cancers worldwide. Studies have shown that small nucleolar RNAs (snoRNAs) play important roles in several cancers. In this study, we analyzed the snoRNAs that were differentially expressed between gastric tumors and normal tissues, identified survival-associated snoRNAs, and developed an eight-snoRNA signature to predict overall survival of patients with gastric cancer. Furthermore, we explored the clinical significance of the eight signature snoRNAs. The risk biomarker established by the eight snoRNA signature was an independent prognostic factor (hazard ratio = 3.43, 95% confidence interval: 1.93–6.09, *P* = 2.72e-05). Furthermore, we validated the expression pattern of those snoRNAs in different gastric cancer cell lines and 5 paired normal and tumor tissues by using real time quantification PCR. Knocking down U66, one of the eight snoRNAs, inhibited the cell proliferation. In conclusion, we identified an eight-snoRNA risk signature to predict overall survival of gastric cancer patients. Seven of these snoRNAs were associated with clinical features of the disease. Knocking down U66 inhibited cell proliferation. These findings provide new clues with prognostic and therapeutic implications in gastric cancer.

## Introduction

Gastric cancer (GC) is one of the leading causes of cancer-related death around the world and is the second and third most common cancer in men and women, respectively, in China (1). Many factors contribute to the genesis of GC such as methylation of genes (2), copy number variation (3, 4), positive family history of GC, cigarette smoking, and low consumption of fruits (5). Compared with other cancers, the prognosis is poor with a 5-year survival rate less than 40% (6). This is in part because there are no strong genetic biomarkers for GC. As a result, new biomarkers to improve the predictive value of the incidence and prognosis of GC are desperately needed. Such biomarkers could help to understand cancer pathogenesis and provide personalized treatment.

Small nucleolar RNAs (snoRNAs) are a class of small non-coding RNA molecules, 60–300 base pairs in length. They are encoded predominantly in introns of host genes in vertebrates, and guide site-specific chemical modifications of ribosomes, transfer RNAs, and small nuclear RNAs. There are two main classes of snoRNAs based on sequence motifs and secondary structural elements: C/D box and H/ACA box snoRNAs. Because of advances in next generation sequencing and experimental and computational approaches, many snoRNAs and their functions are being identified. However, there are many orphan snoRNAs that have no known targets or specific functions.

Recent studies described snoRNAs that displayed unique characteristics and expression patterns, as well as interacting with corresponding protein partners and performing various functions. Increasing attention is being paid to cancer-related snoRNAs. For example, growth arrest-specific transcript 5-associated snoRNAs correlated with TP53 expression and DNA damage in colorectal cancer (7). In addition, C/D-box snoRNAs are associated with metastatic progression and malignant transformation in prostate cancer (8). Finally, snoRNAs and fibrillarin, an enzymatic small nucleolar ribonucleoprotein, are frequently upregulated in human breast and prostate cancers, and those upregulated snoRNAs play crucial roles in tumorigenicity both *in vivo* and *in vitro* (9).

Overall, the results of these studies support the importance of snoRNAs in cell biological processes. Understanding the molecular mechanisms underlying the development of GC is essential for cancer diagnosis and therapy. However, the functions of snoRNAs in GC remain elusive. In the current study, we identified differentially expressed snoRNAs, developed a snoRNA-based signature to predict overall survival of patients with GC, and explored the potential clinical significance of snoRNAs.

## Materials and Methods

### Data Collection and Processing

**Figure 1:**
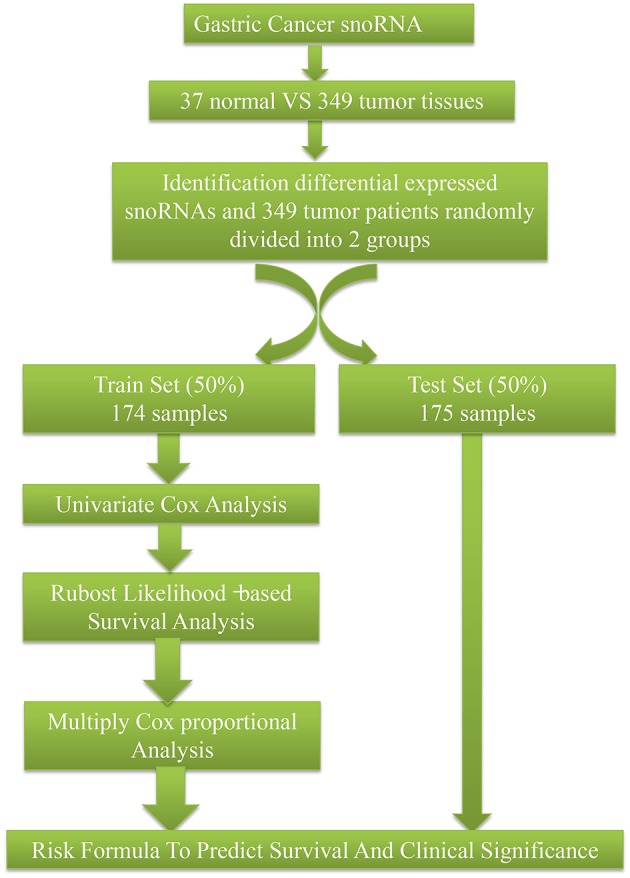
The main workflow of this study.

SnoRNA expression data (fragments per million kilobases for each snoRNA) were downloaded from SNORic, a website used to explore snoRNAs in different cancers with data from The Cancer Genome Atlas (10), and corresponding clinical follow-up data from The Cancer Genome Atlas data portal. Figure 1 shows the main workflow. We filtered snoRNAs that were expressed at least 30% of samples and removed patients without complete clinical information. In total, 37 normal tissues and 349 tumor samples were included in this study. These tumor samples were assigned randomly into a training set (50%, 174), that was used to develop a risk signature and a test set (50%, 175), to verify the performance of the snoRNA signature. There was no significant difference in demographic characteristics between the training and test sets. The basic clinical information is shown in Table 1. Overall, 324 snoRNA profiles were acquired for all patients. This study meets the publication guideline of TCGA (https://www.cancer.gov/about-nci/organization/ccg/research/structural-genomics/tcga/using-tcga/citing-tcga). As the data used in the study was obtained from public datasets, there was no need for additional written consent.

**Table 1:** Clinical covariates for included patients.

**Covariate**		**Total set *n* = 349**	**Training set *n* = 174**	**Testing set *n* = 175**	***P*-value^#^**
Age, *n*	≥65	202	97	105	*P* = 0.421
	<65	147	77	70	
Gender	Male	134	67	67	*P* = 0.966
	Female	215	107	108	
Pathological stage, *n*	I + II	156	77	79	*P* = 0.867
	III + IV	193	97	96	

# *x^2^-test*.

### Identification of Differentially Expressed and Survival-Related snoRNAs

The presence of snoRNAs that were differentially expressed between normal and tumor tissues was analyzed by the *t*-test. A univariate COX proportional regression was applied to identify survival-related snoRNAs. The 30 snoRNAs with the lowest *P*-values were put into a robust likelihood model by the rbsurvR package (11). Firstly, the model placed N^*^(1 – p) samples randomly into the internal training set, and N^*^p samples into the validation set. Here, we chose *p* = 1/2. Secondly, the model placed a snoRNA into the training set and calculated the parameter for this snoRNA. Then the logLik for each snoRNA was evaluated with the above parameter, including validation in the internal validation samples. Finally, this model computed the Akaike information criterion, which is an estimator of the relative quality of statistical models for a given data set. We chose the optimal model with the smallest Akaike information criterion. *P* < 0.05 was considered statistically significant.

### Establishment and Validation of the Risk Formula

SnoRNAs were chosen with the criteria mentioned above and a multivariate Cox analysis was used to calculate coefficients in the training set to establish risk formula by which a risk score for each sample was calculated. All patients were classified into two different groups (high and low risk) based on the median of the risk score. The Kaplan-Meier method and log-rank test were applied to analyze the overall survival of the two groups by using the R package of survival (12, 13). To evaluate the predictive value of the risk model, a receiver operating characteristic (ROC) curve was constructed using the R package of survivalROC (14). Figures were plotted by ggplot2 (15) and ggfortify (16).

### Exploration of the Clinical Significance

We analyzed the expression patterns of snoRNAs that were identified by the risk formula signature. Clinical correlation [Lauren class molecular (17), neoplasm histologic grade, and pathologic stage subtypes] analyses were obtained from SNORic (10).

### Experiment Validation

Real-time quantitative PCR was used to measure the expression prolife of snoRNAs in five gastric cancer cell lines (SGC-7901, BGC-823, NCI-N87, MGC-803, and AGS) and one normal gastric mucosal cell line (GES-1). The primer sequence of the snoRNAs was presented in Supplementary Table 1. The PCR product was sequenced by Sanger method and blast in NCBI, which indicated seven of eight primers work well (Supplementary Figures S1, S2). We collected five patients' tumor and adjacent tissue from surgical specimens which has been approved by Ethics Committee of our hospital. According the expression profile, we selected U66 to test its function. Small interfering RNA (SiRNA) was used to knock down U66. The effect of U66 on cell proliferation was measured by Cell Counting Kit-8.

## Results

We identified 259 snoRNAs that were differentially expressed in GC compared with normal tissues (Supplementary Table 2). Primarily, we used a univariate COX proportional regression to select survival-related snoRNAs in the training set. The 30 snoRNAs with the lowest *P*-values were used to develop the risk formula to predict overall survival. The risk formula was as follows: (0.0496)^*^(expression of U66) + (−0.0191)^*^(expression of ACA47) + (0.0363)^*^(expression of ACA10) + (−0.1711)^*^(expression of E2) + (0.0650)^*^(expression of SNORA58) + (0.0953)^*^(expression of HBII-316) + (−0.4749)^*^(U70) + (−0.2352)^*^(expression of U8).

**Figure 2:**
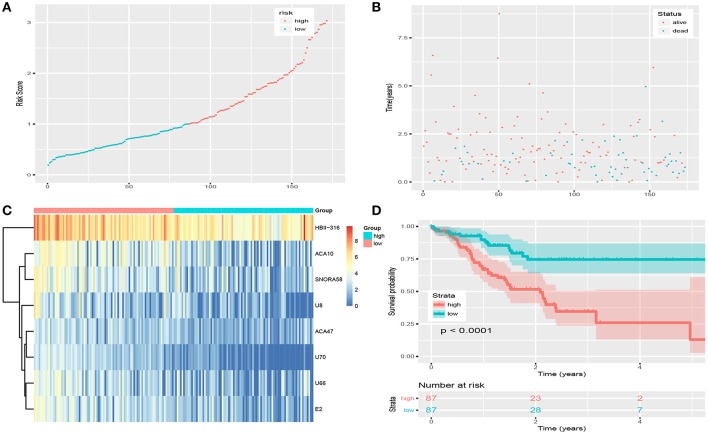
Risk score of snoRNAs-based signature in training set. **(A)** The risk score of patients in train set based on risk signature. **(B)** The distribution of patients' survival status and time. **(C)** The expression profile of eight snoRNAs in train set. **(D)** Survival curve of low-risk group and high-risk group based on median risk score via Kaplan-Meier method.

**Figure 3:**
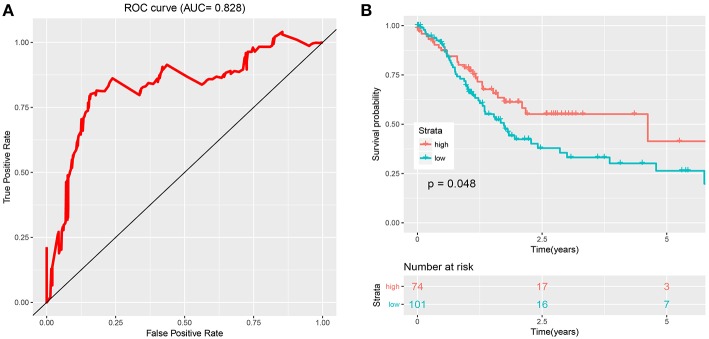
Evaluation and validation of the risk signature. **(A)** The ROC curve of eight-snoRNAs' model. **(B)** The survival curve of two groups (low risk and high risk) based on optimal cutoff in testing set.

Figures 2A,C show details of the normal and GC tissue groups based on risk score calculated by the risk formula. Survival analysis revealed a significant difference between the two groups (Figures 2B,D). The high risk group had significantly shorter overall survival than the low risk group (*p* < 0.0001). The hazard ratio of this risk formula as a prognostic biomarker, was 3.43 (95% confidence interval: 1.93–6.09, *P* = 2.72e-05). The area under the ROC curve (AUC) of the risk formula was up to 0.828 (Figure 3A).

The optimal cutoff was identified as 0.94 with the best Youden's index: 0.64 (sensitivity: 80.1%, specificity: 84.1%). With this cutoff, patients in the test set were divided into two groups (high risk and low risk). Kaplan-Meier curves of the validation data set indicated a significantly prolonged survival time in low-risk compared to high-risk patients (Figure 3B; *P* < 0.05). Results from the test set were highly consistent with results from the training set. This suggested that the snoRNA-based signature had good performance in predicting overall survival.

**Figure 4:**
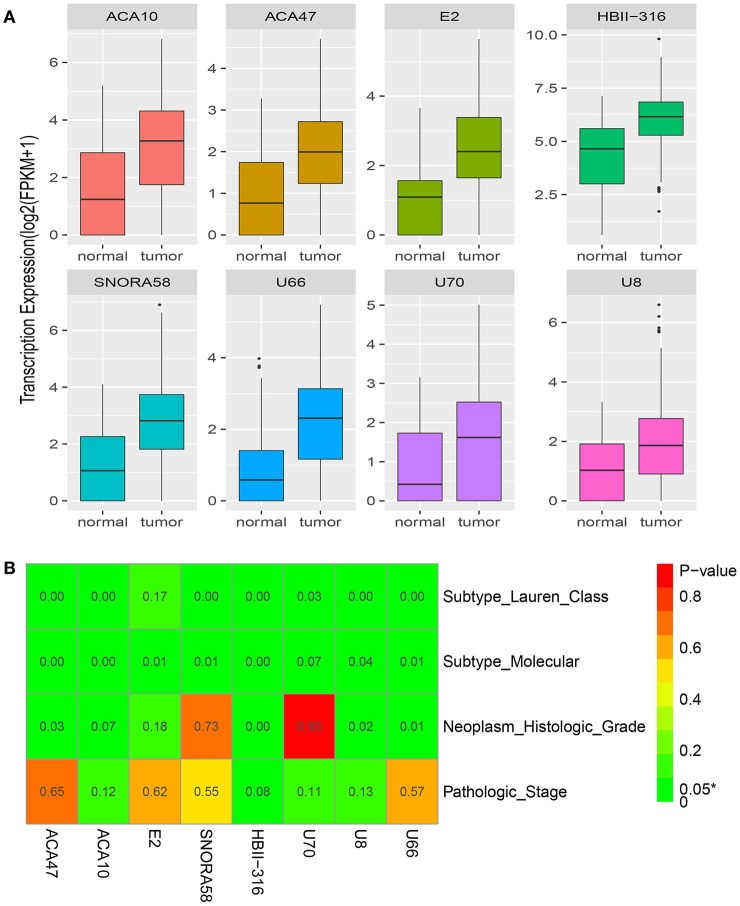
Clinical significance of the eight snoRNAs. **(A)** The expression profile of eight snoRNAs between normal and tumor tissues. **(B)** The correlation between clinical features and the eight snoRNAs.

Figure 4A shows the snoRNA expression patterns between normal and tumor tissues. We found eight snoRNAs (ACA47, E2, ACA10, SNORA58, HBII-316, U70, U8, and U66) that were upregulated in tumor compared with normal tissues (*P* < 0.05). Furthermore, there was a correlation between the eight snoRNAs and clinical factors (Figure 4B). Seven (ACA47, ACA10, SNORA58, HBII-316, U70, U8, and U66) of the eight snoRNAs were associated with the Lauren classification that divides GC into three types: intestinal, diffuse, and mixed. Seven (ACA47, E2, ACA10, SNORA58, HBII-316, U8, and U66) of eight snoRNAs correlated with the molecular subtype (18). Four (ACA47, HBII-316, U8, and U66) of eight snoRNAs were related with the neoplasm histologic grade. However, none of these eight snoRNAs were statistically correlated with pathologic stage.

**Figure 5:**
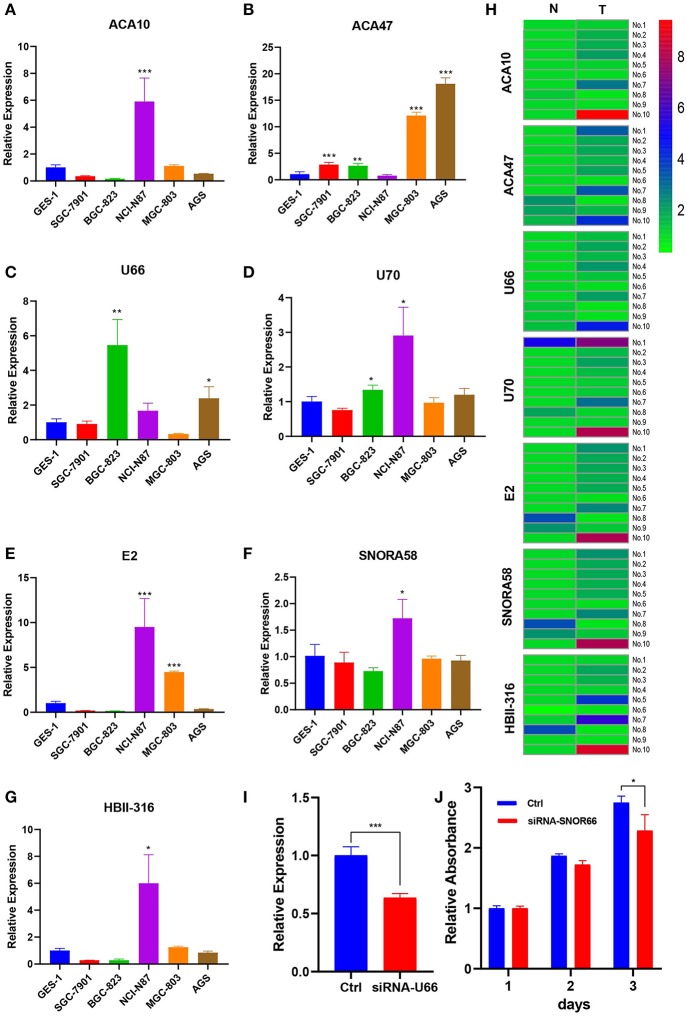
Expression profile of snoRNAs in cell lines and paired tissues. **(A–G)** Expression profile of snoRNAs upregulated in certain cell lines. **(H)** SnoRNAs were mainly upregulated in five patient samples. **(I)** U66 was knocking down by siRNA. **(J)** Knocking down U66 inhibited cell proliferation. *p < 0.05; **p < 0.01; ***p < 0.001.

Those seven (ACA47, E2, ACA10, SNORA58, HBII-316, U70, and U66) of eight snoRNAs were detected in cell lines. Figures 5A–G showed the expression profile of the snoRNAs. Compared with normal tissue, the expression of seven snoRNAs was upregulated in patients (Figure 5H). The effect of siRNA of U66 was validated in NCI-N87 (Figure 5I). Knocking down U66 inhibited the cell proliferation of NCI-N87 (Figure 5J).

## Discussion

Because of advances in high throughput sequencing, numerous snoRNAs have been identified and are emerging as important RNAs, thereby attracting the attention of researchers. Studies have shown that some snoRNAs play important roles in biological processes, and dysfunction of snoRNAs may lead to oncogenesis (19). These studies also indicated that snoRNAs could serve as biomarkers in several diseases, including cancers (20).

In the current study, we used a risk-based formula through multivariate Cox coefficients to identify eight snoRNAs that were differentially expressed between normal and GC tissues (ACA47, E2, ACA10, SNORA58, HBII-316, U70, U8, and U66). The high risk group classified by the risk score had a shorter survival time than the low risk group. These results suggested the eight-snoRNA signature had potential predictive value, and may play a crucial role in the molecular pathogenesis, progression, and prognosis of GC.

The AUC of the ROC was up to 0.828. This indicated that this risk signature had good performance to predict the overall survival of GC patients. Furthermore, Kaplan-Meier survival analysis demonstrated that patients in the high risk group had a shorter overall survival time than those in the low risk group. Thus, the risk biomarker established by the eight-snoRNA signature served as an independent prognostic factor (hazard ratio = 3.43, 95% confidence interval: 1.93–6.09, *P* = 2.72e-05). To our knowledge, this is the first time a risk formula signature was developed using a snoRNA expression profile to predict overall survival of GC patients. These results imply that this risk formula may be used as a novel biomarker.

We also explored the clinical significance of snoRNAs in GC. A clinical features association analysis revealed that seven snoRNAs correlated with the Lauren classification. This classification places GC into three histological subtypes, and has an important influence on prognosis in GC because survival varies depending upon the subtype (21). Seven snoRNAs also correlated with the molecular subtype that classifies GC into four groups: Epstein-Barr virus positive tumors, microsatellite unstable tumors, genomically stable tumors, and tumors with chromosomal instability (17, 18). Therefore, upregulated snoRNAs may be involved in important biological processes such as microsatellite instability, genomic stability, and chromosomal instability. Although none of the eight snoRNAs correlated statistically with pathologic stage, they may still play important roles in GC biological processes.

This work provides some new clues with clinical implications for the development of novel prognostic factors in GC. Although these eight prognostic snoRNAs have not been investigated previously in cancers, the results indicate that they may be involved in tumorigenesis. We validated seven of eight snoRNAs expression profile both in cell lines and patients' tissue. We validated the function of one snoRNA, U66, which may promote cell proliferation.

A limitation of this study was the analysis of only a single data set because other snoRNA datasets are lacking. Thus, further experiments and more samples are needed to validate these findings.

## Conclusions

In conclusion, 259 differentially expressed snoRNAs were identified and used to develop an eight-snoRNA signature from prognosis-related snoRNAs to predict the overall survival of GC with an AUC up to 0.828. We also explored the potential clinical significance of the eight snoRNAs and found that most were correlated with clinical factors. Overall these results provide further insight into the role of snoRNAs in GC. Further experiment indicated that U66 may promote cell proliferation. Importantly, they may have potential prognostic and therapeutic implications for GC, and serve as predictive biomarkers of overall survival.

## Data Availability

Publicly available datasets were analyzed in this study. This data can be found here: http://bioinfo.life.hust.edu.cn.

## Author Contributions

XW, MX, YY, YK, and PL performed the research study and collected the data. XW and MX analyzed the data. BJ and HL designed the research study. XW, MX, and BJ wrote the paper. WZ and PL prepared all the tables. All authors reviewed the manuscript and contributed significantly to this work. In addition, all authors have read and approved the manuscript.

### Conflict of Interest Statement

The authors declare that the research was conducted in the absence of any commercial or financial relationships that could be construed as a potential conflict of interest.
