# Supplementary material for: Identification of Eight Small Nucleolar RNAs as Survival Biomarkers and Their Clinical Significance in Gastric Cancer
Source: Front Oncol. 2019 Sep 6;9:788. doi: 10.3389/fonc.2019.00788 (PMC6747046; doi:10.3389/fonc.2019.00788)
Supplement: Supplementary file 1 [file Data_Sheet_1.pdf]

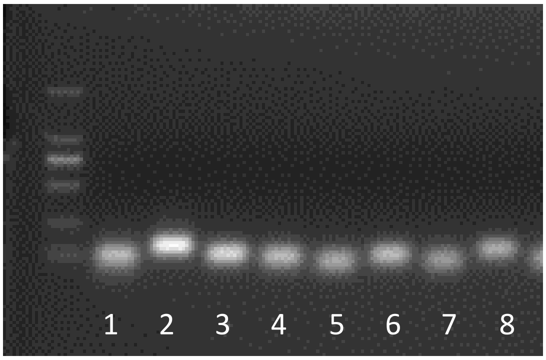

Figure S1. PCR product in GES-1 cell line.

1. U66
2. ACA47
3. ACA10
4. E2
5. SNORA58
6. HBII-316(SNORD92)
7. U70
8. GAPDH

Figure S2. Product of PCR was sequenced and blast in NCBI.

A.U66(SNORA66)

Mouse over to see the title, click to show alignments

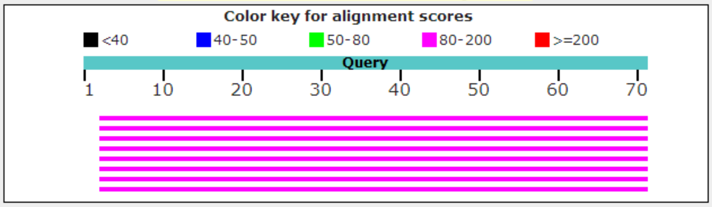

Descriptions

Sequences producing significant alignments:

Select: All None Selected:0

Alignments Download GenBank Graphics Distance tree of results

Description	Max Score	Total Score	Query Cover	E value	Per. Ident	Accession
PREDICTED: Pan paniscus small nucleolar RNA SNORA66 (LOC112438427), ncRNA	122	122	97%	2e-25	98.57%	XR_003026849.1
PREDICTED: Pan troglodytes small nucleolar RNA SNORA66 (LOC112207292), ncRNA	122	122	97%	2e-25	98.57%	XR_002941724.1
Homo sapiens small nucleolar RNA, H/ACA box 66 (SNORA66), small nucleolar RNA	122	122	97%	2e-25	98.57%	NR_002444.2
PREDICTED: Pongo abelii small nucleolar RNA SNORA66 (LOC112131317), ncRNA	117	117	97%	1e-23	97.14%	XR_002913413.1
PREDICTED: Theropithecus gelada ribosomal protein L5 (RPL5), transcript variant X1, mRNA	104	104	97%	9e-20	94.29%	XM_025353509.1
PREDICTED: Macaca nemestrina small nucleolar RNA SNORA66 (LOC112427372), ncRNA	104	104	97%	9e-20	94.29%	XR_003018898.1
PREDICTED: Piliocolobus tephrosceles small nucleolar RNA SNORA66 (LOC111555194), ncRNA	104	104	97%	9e-20	94.29%	XR_002735452.1

B. ACA47

Mouse over to see the title, click to show alignments

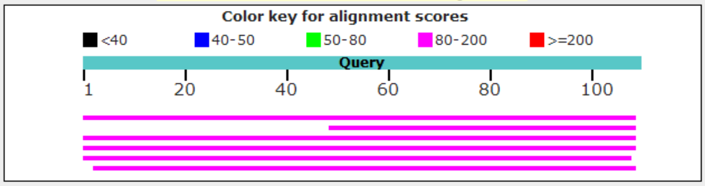

escriptions

Sequences producing significant alignments:

Select: All None Selected:0

Alignments Download GenBank Graphics Distance tree of results

Description	Max Score	Total Score	Query Cover	E value	Per. Ident	Accession
Transcripts						
Homo sapiens small Cajal body-specific RNA 16 (SCARNA16), guide RNA	193	193	99%	2e-47	99.07%	NR_003013.1
Homo sapiens microRNA 6516 (MIR6516), microRNA	111	111	55%	6e-23	100.00%	NR[illegible]

C. SNORA10

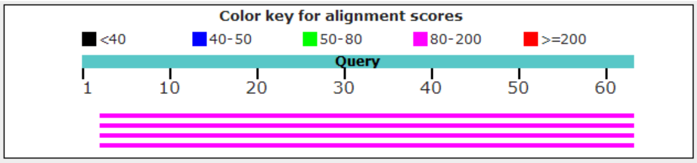

Descriptions

Sequences producing significant alignments:

Select: All None Selected:0

Alignments Download GenBank Graphics Distance tree of results

Description	Max Score	Total Score	Query Cover	E value	Per. Ident	Accession
**Transcripts**						
Homo sapiens small nucleolar RNA, H/ACA box 10 (SNORA10), small nucleolar RNA	113	113	96%	8e-24	100.00%	NR_002327.1

D. E2(SNORA62)

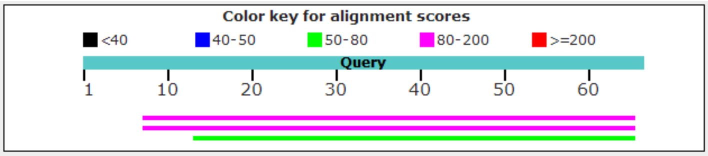

Descriptions

Sequences producing significant alignments:

Select: All None Selected:0

Alignments Download GenBank Graphics Distance tree of results

Description	Max Score	Total Score	Query Cover	E value	Per. Ident	Accession
**Transcripts**						
Homo sapiens small nucleolar RNA, H/ACA box 62 (SNORA62), small nucleolar RNA	102	102	87%	2e-20	98.31%	NR_002324.1

E. SNORA58

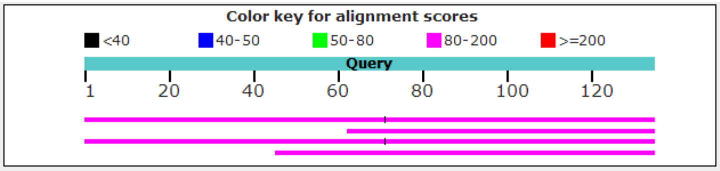

Descriptions

Sequences producing significant alignments:

Select: All None Selected:0

Alignments Download GenBank Graphics Distance tree of results

	Description	Max Score	Total Score	Query Cover	E value	Per. Ident	Accession
	Transcripts						
☐	Homo sapiens small nucleolar RNA, H/ACA box 58 (SNORA58), small nucleolar RNA	145	264	100%	8e-33	100.00%	NR_002985.2
☐	Homo sapiens small nucleolar RNA, H/ACA box 58B (SNORA58B), small nucleolar RNA	108	108	54%	1e-21	93.15%	NR_145714.1

F. HBII-316(SNORD92)

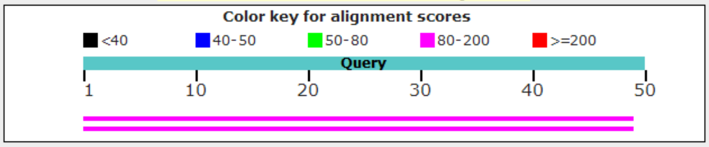

Descriptions

Sequences producing significant alignments:

Select: All None Selected:0

Alignments Download GenBank Graphics Distance tree of results

	Description	Max Score	Total Score	Query Cover	E value	Per. Ident	Accession
	Transcripts						
☐	Homo sapiens small nucleolar RNA, C/D box 92 (SNORD92), small nucleolar RNA	82.4	82.4	98%	1e-14	96.08%	NR_003074.1

G. SNOR70

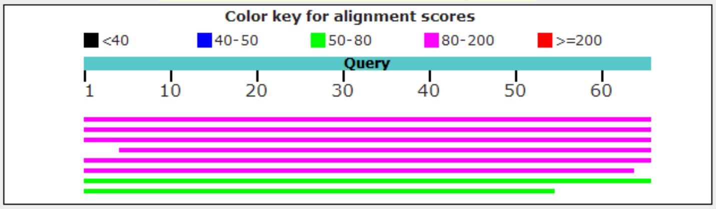

Descriptions

Sequences producing significant alignments:

Select: All None Selected:0

Alignments Download GenBank Graphics Distance tree of results

	Description	Max Score	Total Score	Query Cover	E value	Per. Ident	Accession
	**Transcripts**						
☐	Homo sapiens small nucleolar RNA, H/ACA box 70 (SNORA70), small nucleolar RNA	113	113	100%	8e-24	98.46%	NR_000011.1
☐	Homo sapiens small nucleolar RNA, H/ACA box 70I (SNORA70I), small nucleolar RNA	102	102	100%	2e-20	95.38%	NR_145773.1
